# Dramatic polarization in genitourinary expert opinions regarding the clinical utility of positron emission tomography (PET) imaging in prostate cancer

**DOI:** 10.1590/S1677-5538.IBJU.2018.0208

**Published:** 2019

**Authors:** Kiri A. Sandler, Shearwood McClelland, Catherine Degnin, Yiyi Chen, Timur Mitin

**Affiliations:** 1 Department of Radiation Oncology, University of California at Los Angeles, Los Angeles, CA, USA; 2 Department of Radiation Oncology, Indiana University School of Medicine, Indianapolis, IN, USA; 3 Department of Radiation Medicine, Oregon Health and Science University, Portland, OR, USA; 4 Biostatistics Shared Resource, Oregon Health and Science University, Portland OR, USA

**Keywords:** Prostatic Neoplasms, Positron-Emission Tomography, Radiotherapy

## Abstract

**Objectives:**

To ascertain the opinions of North American genitourinary (GU) experts regarding inclusion of technologies such as prostate - specific membrane antigen (PSMA) and C – 11 choline positron emission tomography (PET) into routine practice.

**Materials and Methods:**

A survey was distributed to North American GU experts. Questions pertained to the role of PSMA and C – 11 PET in PCa management. Participants were categorized as “supporters” or “opponents” of incorporation of novel imaging techniques. Opinions were correlated with practice patterns.

**Results:**

Response rate was 54% and we analyzed 42 radiation oncologist respondents. 17 participants (40%) have been in practice for > 20 years and 38 (90%) practice at an academic center. 24 (57%) were supporters of PSMA and 29 (69%) were supporters of C – 11. Supporters were more likely to treat pelvic nodes (88% vs. 56%, p < 01) and trended to be more likely to treat patients with moderate or extreme hypofractionation (58% vs. 28%, p = 065). Supporters trended to be more likely to offer brachytherapy boost (55% vs. 23%, p = 09), favor initial observation and early salvage over adjuvant radiation (77% vs. 55%, p = 09), and to consider themselves expert brachytherapists (69% vs. 39%, p = 09).

**Conclusions:**

There is a polarization among GU radiation oncology experts regarding novel imaging techniques. A correlation emerged between support of novel imaging and adoption of treatment approaches that are clinically superior or less expensive. Pre - existing biases among GU experts on national treatment - decision panels and leaders of cooperative group studies may affect the design of future studies and influence the adoption of these technologies in clinical practice.

## INTRODUCTION

Prostate cancer (PCa) is the most common malignancy in men. Primary management strategies for PCa include surgery or radiation therapy (RT). Despite modern surgical and radiation techniques, biochemical recurrence (BCR) rates remain relatively high, approaching 20 – 40% regardless of management technique (1-3). The ability to detect ever lower levels of prostate-specific antigen (PSA) has improved the ability to identify BCR, but localizing the cells that are producing PSA is not straightforward. Imaging in the setting of recurrent prostate cancer is notoriously challenging. Commonly used imaging modalities such as computed tomography (CT) and fluorodeoxyglucose positron - emission topography (FDG - PET) have low sensitivity for local recurrence and small metastases (4). Even technetium – 99 bone scans, the gold standard for detecting osseous metastases in men with prostate cancer, perform poorly in patients with low PSA. A positive bone scan in the absence of high - risk factors is more likely to be a false positive (5). Unfortunately, once the PSA is high enough for detection by traditional methods, the window for clinical intervention may have closed.

These challenges have led to an interest in radionuclides that can identify an actionable recurrence earlier in the disease course. Novel PET tracers such as C – 11 choline and gallium – 68 labelled prostate specific membrane antigen (PSMA) have shown promise in early detection of small foci of recurrent disease, both local and distant, in patients with BCR (6-8). Remarkably, PSMA has been able to localize recurrent disease (for lymph node involvement) with sensitivity and specificity of 94% and 99%, respectively, even at a low PSA (9). C – 11 choline has shown similarly encouraging results, with recurrences detected at PSA levels < 1 ng / mL (10, 11).

A recent review of the clinical utility of prostate cancer-specific PET radiotracers has indicated that PSMA is the most sensitive of the currently clinically evaluated prostate radiotracers (12).

The National Comprehensive Cancer Network (NCCN) in 2017 recommended consideration of C – 11 PET in the setting of detectable PSA after prostatectomy, BCR after definitive RT, and in patients without metastases who are on androgen - deprivation therapy (ADT) with a rising PSA. It did not make recommendations regarding the use of PSMA. What is not known, however, is whether novel imaging techniques are being adopted into routine practice. Genitourinary (GU) oncology experts, such as those who serve on decision - making committees of cooperative group research organizations, lead the field in evidence - based investigation and adoption of new diagnostic and therapeutic techniques. They also tend to be positioned at large, tertiary - care and academic medical institutions where novel radionuclides are more likely to be available. In this study, we seek to query GU oncology experts to assess the opinions and trends of utilization of novel imaging techniques.

## MATERIALS AND METHODS

### Survey design and deployment

The survey was designed to identify characteristics of each respondent's typical practice patterns, as well as to assess their knowledge about and personal opinions on the role of PSMA and C – 11 choline PET in PCa management. Eighty - eight currently practicing North American GU oncology experts, who serve on cooperative group research organizations such as NRG Oncology, were contacted by email and invited to complete our survey. The survey was designed and hosted by Research Electronic Data Capture (REDCap) (13). The survey contained screening questions to ensure respondents were currently practicing, not in training, and specialize in GU oncology. The study was approved by the institutional review board. A copy of the survey is available in the Supplementary Material.

### Selection of practice pattern questions

Topics for inclusion were selected based on their controversy within the field of radiation oncology. Many of the practices in question, despite having been studied in large phase III randomized trials, are points of divisiveness amongst radiation oncologists and serve as branch points for practice patterns. For example, moderate hypofractionation has been proven to be noninferior, safe, and more economical than standard fractionation (14), however many experts are still slow to adopt this practice. Pelvic lymph node irradiation is notoriously controversial in radiation oncology (15), and adjuvant vs. early salvage postoperative radiation is an area of evolving study (16).

### Statistical analysis

Descriptive statistical analysis was conducted to describe characteristics of respondents. Participants were categorized as “supporters” or “opponents” of incorporation of novel imaging techniques into routine practice, with support of C – 11 choline PET and PSMA PET analyzed separately. Opinions regarding novel imaging were correlated with practice patterns using Fisher's exact test. McNemar's test was used to test the differences in distribution for supporting C – 11 choline and for supporting PSMA PET among respondents.

## RESULTS

### Respondents

We received 48 responses for a response rate of 54%. For comparative studies, we limited our analysis to 42 radiation oncologist respondents. Seventeen participants (40%) have been in practice for > 20 years and 38 (90%) practice at an academic center. Nineteen participants (45%) see > 20 patients / month in consultation. Twenty - two respondents (52%) see an even balance of intact prostate and post - prostatectomy patients, and 17 (40%) see primarily intact prostate patients. The majority of respondents (35, 83%) perform a digital rectal examination (DRE) prior to treatment and half of respondents felt that DRE changes management. Twenty - five respondents (60%) consider themselves to be expert brachytherapists.

### Practice characteristics

The majority of participants (40, 95%) recommend active surveillance for patients with Gleason 6 disease, and a few (7, 17%) recommend it for Gleason 3 + 4 disease. Most respondents (29, 69%) often treat pelvic lymph nodes in patients with localized high risk disease. There was a fairly even split (55% vs. 45%) between whether respondents recommend adjuvant RT or observation and early salvage RT, respectively, for patients with high risk features. In a similar pattern, 45% of respondents recommended the addition of a brachytherapy boost for patients with high risk disease and no baseline urinary symptoms, while the remaining 55% would recommend external beam radiation and ADT alone. Regarding dose and fractionation, 23 (55%) consider standard fractionation (78 Gy in 2 Gy fractions or equivalent) to be the default for a patient with Gleason 3 + 4 disease. Seventeen (40%) chose moderate hypofractionation (70 Gy in 2.5 Gy fractions or equivalent) as the default, and two (5%) chose stereotactic body radiation therapy (SBRT) / radical hypofractionation (5 – 12 fractions). A summary of practice characteristics is provided in Table-1.

**Table 1:** Practice characteristics.

GU Expert Practice Recommendation (n = 42)		Participants selecting response n (%)
**Active Surveillance (AS)**		
	Recommend AS for GS 6	40 (95)
	Recommend AS for GS 3 + 4	7 (17)
**Management of oligometastatic PCa**		
	Offer SBRT to oligometastatic patient outside of clinical trial	32 (76)
**Prophylactic pelvic irradiation**		
	Treat pelvis in localized high risk patients	29 (69)
**Management of pT3 disease after RP**		
	Recommend adjuvant RT for high risk operative patients	23 (55)
	Recommend observation and early salvage RT for high risk operative patients	19 (45)
**Management of High Risk PCa**		
	Recommend EBRT and ADT	23 (55)
	Recommend brachy boost with EBRT and ADT	19 (45)
**Fractionation scheme for low / intermediate risk**		
	Recommend standard fractionation (i.e. 1.8-2Gy / fx)	23 (55)
	Recommend moderate hypofractionation (i.e. 2.5 – 3Gy / fx)	17 (40)
	Recommend SBRT / radical hypofractionation	2 (5)
**Management of low risk PCa patient who desires treatment**		
	Recommend EBRT	7 (17)
	Recommend brachytherapy	21 (50)
	Recommend either EBRT or brachytherapy	14 (33)

**GU** = genitourinary oncology; **PCa** = prostate cancer; **GS** = Gleason score; **SBRT** = stereotactic body radiation therapy; **RT** = radiation therapy; **ADT** = androgen deprivation therapy

### Novel imaging

Twenty - seven respondents (60%) were already aware that the NCCN recommends consideration of C – 11 PET for patients with prostate cancer. The NCCN guidelines were then presented to respondents. Following this, a series of questions was posed to ascertain whether respondents agreed with the NCCN recommendations or whether they felt C – 11 or PSMA PET should be recommended in more or fewer situations than the current guidelines dictate. Opponents were defined as those who answered that novel imaging techniques should not be recommended by the NCCN in any situations due to a lack of evidence. Supporters were defined as those who answered that novel imaging should be recommended in at least some scenarios. Twenty - four (57%) were supporters of PSMA PET and 29 (69%) were supporters of C – 11 PET. Regarding the comparison between the two techniques, the majority (27, 66%) feel that there is not enough evidence to know whether C – 11 or PSMA PET is more effective. However, some respondents did rank one test above the other, with 27% and 5% answering PSMA PET and C – 11 PET, respectively.

**Figure 1:**
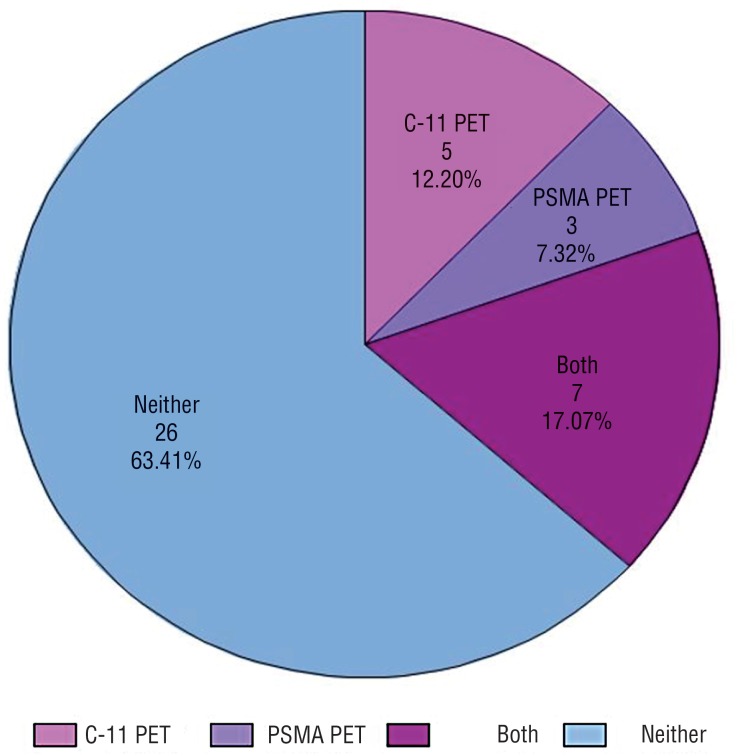
Pie chart demonstrating the number of respondents who have at their institution C – 11 PET, PSMA PET, both of these, or neither.

**Figure 2:**
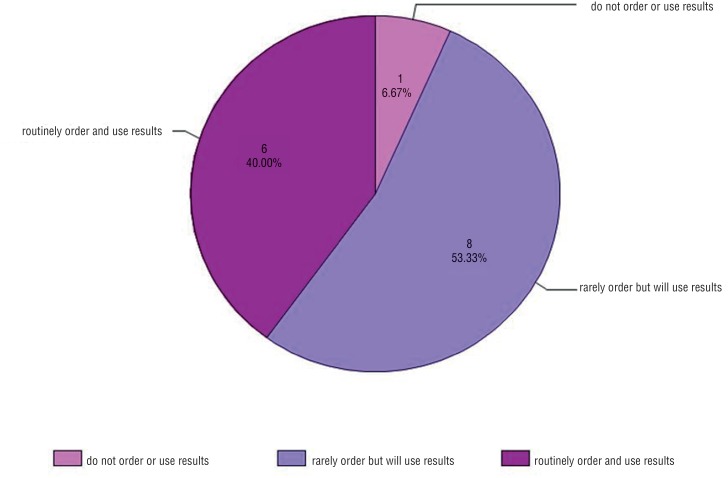
Of respondents who answered they have C – 11 PET, PSMA PET, or both available at their institution, what is the current practice for their use?

**Figure 3:**
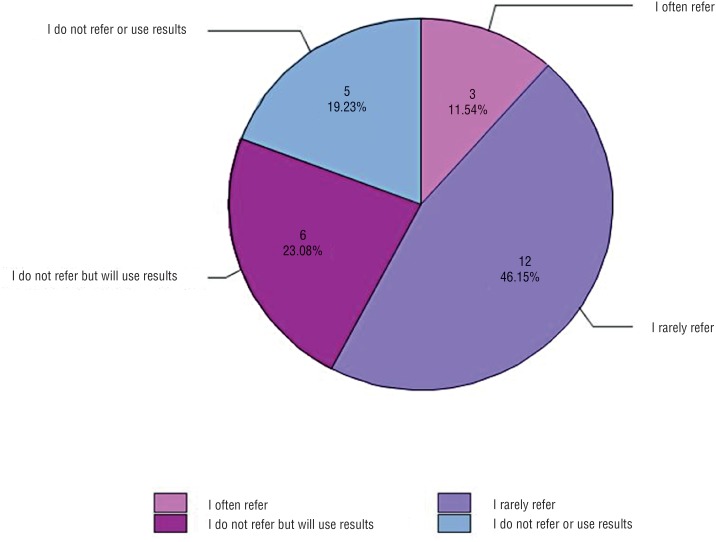
Of respondents who answered they do not have either C – 11 PET or PSMA PET available at their institution, what is the current practice for referral?

Even in a group of experts who primarily practice at academic centers, the majority of respondents (26, 63%) do not have either C – 11 or PSMA PET available at their institution (Figure-1). Of those who do have the tests available (Figure-2), 6 (40%) routinely order them for patients, and 8 (53%) order them on rare occasion. Of those who do not have the tests available (Figure-3), 15 (58%) refer patients to centers capable of performing them. Six (23%) do not refer, but will use the results to guide decision - making if the patient previously underwent imaging. The remaining 19% do not refer and do not use the tests to guide decision - making. When asked for reasons why they do not order PSMA or C – 11 PET more often, providers most frequently answered availability (31, 74%). The second most common reasons were cost (18, 43%) and lack of evidence (15, 36%). Seven respondents (17%) answered that they do not order the scans because they are unsure how to interpret them.

### Patterns of practice in novel imaging supporters

Supporters of PSMA PET were more likely than opponents of PSMA PET to routinely treat pelvic nodes in patients with high risk PCa (88% vs. 44%, Fisher's exact p < 01) and trended to be more likely to treat patients with low/intermediate PCa with moderate (20 – 28 fractions) or extreme (5 – 15 fractions) hypofractionation (58% vs. 28%, Fisher's Exact p = 065). Supporters of C – 11 PET trended to be more likely than opponents of C – 11 PET to offer to patients with high risk PCa brachytherapy boost (55% vs. 23%, Fisher's exact p = 09), favor initial observation and early salvage over adjuvant radiation in patients with high risk features after prostatectomy (55% vs. 23%, Fisher's exact p = 09), and to consider themselves expert brachytherapists (69% vs. 38%, Fisher's Exact p = 09). Table-2 demonstrates the distribution of supporters of C – 11 compared with PSMA PET, revealing an overall trend to either supporting or opposing both agents, with very few respondents showing selectivity for PSMA (2 out of 42) or C – 11 (7 out of 42).

**Table 2:** Distribution of supporters of C – 11 compared with PSMA PET.

	C – 11 Supporter	C – 11 Opponent	Total
PSMA Supporter	22	2	24
PSMA Opponent	7	11	18
**Total**	**29**	**13**	**42**

**McNemar's Test** = p = 0.180

## DISCUSSION

Molecular imaging is a rapidly evolving field that is poised to change the paradigm for how BCR in PCa is managed. However, with the abundance of data revealed by each scan and with new research constantly emerging, it can be challenging to determine if and when these imaging techniques should be employed. In this study, we surveyed a group of highly specialized GU oncology experts who serve on decision - making committees of collaborative group research organizations regarding their opinions and usage of C – 11 choline and PSMA PET. To our knowledge, this study is the first of its kind to ascertain expert opinion on the topic and to correlate those opinions with practice characteristics.

A rising PSA after definitive therapy in a patient who otherwise has no evidence of PCa represents a therapeutic challenge. Practitioners typically use a variety of factors such as PSA level, PSA doubling time, Gleason score, and margin status to predict whether a patient's recurrence is local or distant. While local salvage therapy does improve outcomes and provide cure in some patients (17), men with a persistently elevated PSA or those who develop clinically evident metastases are sentenced to lifelong ADT and its myriad side effects. Treatment of a localized recurrence may have the potential to spare patients from this fate. An emerging area of interest in PCa, among other cancers, is the effort to slow disease progression and potentially offer cure in oligometastatic patients. Though most reports have been from small series of patients, initial results are promising (18). One would surmise that identifying oligometastatic patients earlier with PSMA or C – 11 PET scans could both identify small metastases before they become widespread, as well as offer the benefit of a smaller and more safely ablatable target. In patients who have widespread metastases, PSMA ligand - based radionuclide therapy is another potentially beneficial application of this technology. Small series have shown very encouraging results in terms of disease response and pain relief, with minimal toxicity (19, 20).

Though the NCCN in 2017 made recommendations for the use of C – 11 PET imaging, consensus groups have not released any official guidelines thus far. Providers unfamiliar with C – 11 choline or PSMA PET may struggle with decision - making when presented with these scans. Patients with a low but detectable PSA with a single lesion on PSMA PET may desire definitive treatment, and providers may be eager to offer it, even without pathologic confirmation.

With the available evidence thus far, GU oncology experts are split on whether or not to utilize novel imaging in treatment decision making. Interestingly, this split seems to follow along with a pattern of practice characteristics. Those experts who support practice strictly according to randomized data, such as adding a brachytherapy boost for patients with high risk disease (21, 22) or using moderate hypofractionation (14, 23), also tend to support the use of novel imaging techniques. Supporters of novel imaging techniques were also more likely to treat pelvic lymph nodes in patients with high risk features, a technique which is more aggressive but has not been shown definitively to improve outcomes (24, 25). Perhaps those practitioners who treat patients more aggressively, such as with a brachytherapy boost and pelvic nodal coverage, are more interested in novel imaging studies which would provide them with a therapeutic target in the setting of BCR. Regardless of the logic behind their decision - making, the dichotomy that has emerged among participants in this survey has important implications. Our study suggests that, even in the face of future randomized evidence, GU oncology experts may remain divided over the appropriateness of inclusion of novel imaging in routine practice. It may also delay the implementation of therapy such as PSMA - ligand - based radionuclide therapy. Thus, the inherent biases of experts in large part shape the field of prostate cancer management.

This study has several limitations, including those inherent to all survey studies as previously reported (26). Though we had a substantial response rate of 54%, the absolute number of respondents are small, as we limited our target population only to GU oncology experts who serve on cooperative group research organizations. The small overall sample size also likely contributed to the difficulty with reaching the standard p < 0.05 cutoff for statistical significance for many comparisons. However, many of the differences we report are quite stark, with p values that trend toward significance. An additional limitation is that responses were multiple - choice and may not capture the full range of opinions. Survey fatigue can also result in responses that are not genuine, however there was not an incentive, financial or otherwise, to complete the survey which may improve the rate of legitimate responses. Though the initial results of novel radionuclide studies are promising, further research is still needed to confirm their accuracy. Pathologic confirmation with biopsy of distant metastatic disease or with sentinel lymph node biopsy in the case of radiographically positive lymph nodes (27) will add to the body of literature supporting these techniques. Additionally, the value of early intervention for oligometastatic patients has not yet been fully elucidated. Finally, because only 15 of 41 responders had PET in their facilities (Figure-1), there was a significant potential for origin bias. It is also unclear regarding the familiarity of responders with both imaging methods, which could have influenced the final results of this study.

## CONCLUSIONS

There is a stark polarization among GU radiation oncology experts regarding the use of novel imaging techniques in routine practice. Moreover, there appears to be a correlation between support of novel imaging and adoption of treatment approaches shown in randomized trials to be either clinically superior or less expensive. Pre - existing biases regarding novel imaging among GU experts on national treatment - decision panels and leaders of cooperative group studies may affect the design of future clinical studies and influence the adoption of these technologies in clinical practice. The support of novel imaging techniques among the community of GU experts would open a multitude of possibilities for research in this area.
